# Atypical Cognitive Impairment and Recovery in Two Colorectal Cancer Patients

**DOI:** 10.3390/tomography8030123

**Published:** 2022-06-09

**Authors:** Hui Su Lee, Hwan Ho Jo, Ko Woon Kim, Byoung-Soo Shin, Hyun Goo Kang

**Affiliations:** 1 Medical School, Jeonbuk National University, Jeonju 54907, Korea; heesu9843@jbnu.ac.kr (H.S.L.); jhh503@jbnu.ac.kr (H.H.J.); 2 Department of Neurology, Jeonbuk National University Medical School and Hospital, 20 Geonji-ro, Deokjin-gu, Jeonju 54907, Korea; kowoonkim@jbnu.ac.kr (K.W.K.); sbsoo@jbnu.ac.kr (B.-S.S.); 3 Research Institute of Clinical Medicine of Jeonbuk National University—Biomedical Research Institute of Jeonbuk National University Hospital, 20 Geonji-ro, Deokjin-gu, Jeonju 54907, Korea

**Keywords:** CD8-positive T-lymphocytes, colorectal neoplasms, gastrointestinal microbiome, limbic encephalitis

## Abstract

Cognitive impairment in cancer patients can be caused by various factors; in approximately 30% of cancer patients, the symptoms appear before starting treatment. Paraneoplastic limbic encephalitis (PLE) is a rare disease associated with an autoimmune response, and is characterized by memory loss, depression, and personality changes; it is one of the potential causes of cognitive dysfunction in cancer patients. Two patients were previously diagnosed with mild cognitive impairment and maintained clinical stability; after suffering a rapid change in personality and sudden cognitive decline, colorectal cancer was diagnosed within a few months. The patients did not meet the diagnostic criteria for PLE in several tests. The symptoms improved after the underlying cancer was treated, and the patients returned to their previous stable state. Sudden cognitive impairment may appear as an early cancer symptom, and PLE is considered an atypical cause for these symptoms. However, in patients with unexplained PLE-like symptoms who do not meet the diagnostic criteria for PLE, probable etiologies to be considered are the gut–brain connection, CD8^+^ T-cell-mediated limbic encephalitis, and somatic mutations in dementia-related genes. Currently, few studies have investigated these symptoms, and further research will offer significant therapeutic strategies for cognitive impairment in cancer patients.

## 1. Introduction

Cognitive impairment is a symptom commonly observed in cancer patients. Even when not severe, it is important to accurately diagnose it and manage its causes, because it can affect social relationships, treatment intention, and quality of life. Various factors can cause cognitive impairment in these patients: direct brain lesions, such as brain metastasis of primary cancer; chemotherapy; opioid analgesic administration; head and neck radiation therapy; side effects of immunotherapy (e.g., steroids); and metabolic abnormalities accompanying the patient’s general condition [1]. Although cognitive impairment owing to a paraneoplastic syndrome is not frequent, it should be considered an important cause in the case of cognitive impairment in cancer patients.

Paraneoplastic limbic encephalitis (PLE) is a disease that occurs when tumor-derived autoantibodies, such as anti-Hu, anti-Yo, anti-Ma2, and anti-N-methyl D-aspartate (NMDA) receptor (anti-NMDAR), affect the limbic system. It is characterized by cognitive and behavioral symptoms, including memory loss, personality change, emotional irritability, and dementia [2]. Almost 80% of the neurological disorders caused by this paraneoplastic syndrome manifest symptoms before the cancer is diagnosed [3]. Since PLE patients have a rapid decline in memory and cognitive abilities, usually within three months, a differential diagnosis of rapidly progressive dementia must be considered. The prognosis is generally poor, and is related to the type of autoantibody, the patient’s comorbidities, and the cancer stage [3].

We present two cases of patients with mild cognitive impairment, who were stable for several years, showed sudden cognitive decline and rapid personality changes, and were diagnosed with cancer shortly after. Both patients showed PLE-like symptoms; however, their disease progression was different from general cancer-related cognitive impairment; therefore, we report these cases with a literature review.

## 2. Case Presentation

### 2.1. Case 1

A 68-year-old man visited our hospital complaining of sudden cognitive decline, extreme depression, and intermittent increases in violence, which had started two months prior. At the time of admission, the patient could not determine the current year and season and had recently developed an unwillingness to wash, despite being usually clean. There were no underlying diseases such as hypertension or diabetes. He had been diagnosed with mild cognitive impairment four years before; however, he had previously obtained a score of 21 or higher in the annual Korean Mini Mental-Status Examination (K-MMSE), showing that his memory and cognitive functions were sufficiently maintained. When he visited the hospital, his K-MMSE score was 8, a sharp decrease from 22 the previous year. During the Seoul Neuropsychological Screening Battery (SNSB) test, abnormal behaviors were reported, such as irritability and anger, and a deterioration in memory, attention, spatiotemporal ability, and frontal/executive function. There were no specific findings on brain magnetic resonance imaging (MRI). The cerebrospinal fluid (CSF) was clear, with a pressure of 14 cmH_2_O, a white blood cell count of 3/mm^3^, a glucose level of 68.0 mg/dL, and a slightly elevated protein level of 79.1 mg/dL. No specific abnormalities were observed in the CSF cytology examination. The blood chemistry test showed a leukocyte count of 7610/mm^3^, a Na^+^ level of 133 mEq/L, and a K^+^ level of 4.4 mEq/L, suggesting no metabolic abnormalities. The thyroid function test showed a TSH level of 1.564 uIU/mL, and a Free T4 level of 14.86 pmol/L, showing no hormonal abnormalities.

Medication was administered to control the symptoms, without significant improvement. Two months after symptom onset, the patient was diagnosed with rectal cancer on an abdominal computed tomography (CT) performed for fecal blood and weight loss. A histological examination revealed moderately differentiated adenocarcinoma, staged as T3 N0, stage IIA. The patient underwent preoperative concomitant chemoradiation therapy, and antimetabolite-type capecitabine was administered for six weeks. Simultaneously, whole-pelvis intensity-modulated radiation therapy was performed; eight weeks later, he underwent laparoscopic ultra-low anterior resection. All the serological analysis results for paraneoplastic antibodies were negative. After surgery and chemotherapy, his K-MMSE score was 23, showing improved cognitive functions, and his recent symptoms resolved, returning to his previous stable status. Currently, no recurrence is observed on the colonoscopy.

### 2.2. Case 2

An 82-year-old man, previously healthy and active, visited the hospital with a sudden decrease in cognitive function, and aggression, which had started several weeks before his visit. Before these symptoms, he worked in an office, and despite his old age, he was thorough and meticulous in managing his schedule. However, at the time of his visit, he could not remember important appointments, was violent, and frequently quarreled with others. The patient had no other relevant findings. The K-MMSE score at admission was 13, a considerable drop from 26 only four months prior. At the time of the SNSB examination, the patient was not cooperative; however, despite the limitations in obtaining accurate results, he showed alterations in memory, attention, concentration, frontal lobe functions, and executive functions. A brain MRI and electroencephalography (EEG) did not reveal any abnormalities. The CSF was clear, with a pressure of 12 cmH_2_O, a WBC count of 0/mm^3^, a glucose level of 61.0 mg/dL, and a slightly elevated protein level of 74.0 mg/dL. No specific abnormalities were observed in the CSF cytology examination. The blood chemistry test showed a leukocyte count of 5420/mm^3^, a Na^+^ level of 138 mEq/L, and a K^+^ level of 4.27 mEq/L, showing no metabolic abnormalities. The thyroid function test showed a TSH level of 1.142 uIU/mL, and a Free T4 level of 13.24 pmol/L, showing no hormonal abnormalities.

**Figure 1:**
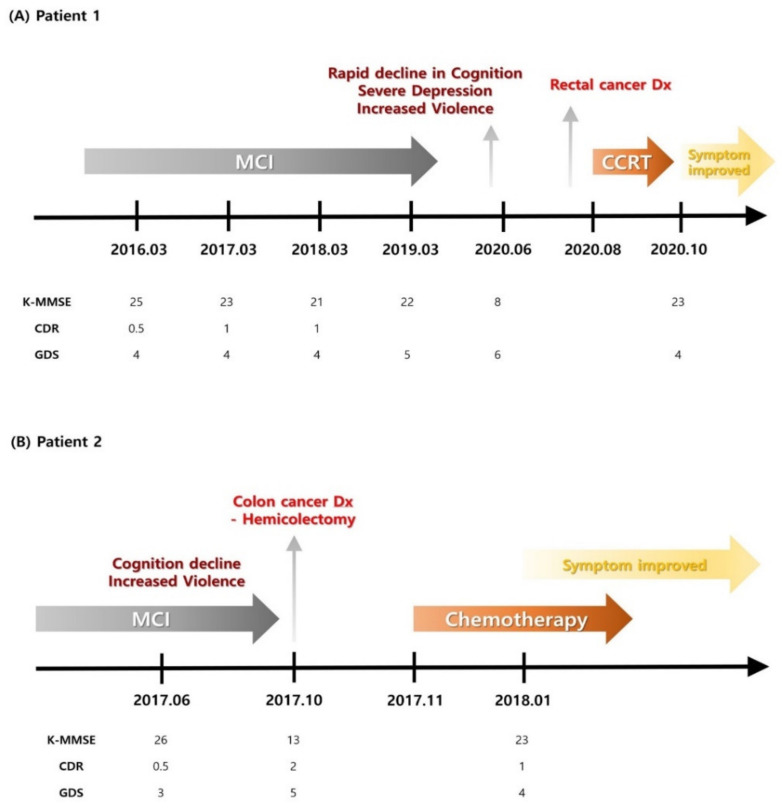
Patients’ timelines. CCRT—concomitant chemoradiation therapy; CDR—clinical dementia rating; Dx—diagnosis; GDS—global deterioration scale; K-MMSE—Korean Mini Mental-Status Examination; MCI—mild cognitive impairment.

Medication was administered to control the symptoms, without significant improvement. An abdominal CT was performed four months after symptom onset due to melena, and an ascending colon tumor with lymph node metastasis was noted. After a laparoscopic right hemicolectomy, a moderately differentiated adenocarcinoma was diagnosed on histological examination, staged as T3 N1b, stage IIIB. The patient underwent modified FOLFOX-6 chemotherapy after the surgery. All the serological analysis results for paraneoplastic antibodies were negative. After surgery and chemotherapy, his K-MMSE score increased to 23, showing improved cognitive function. The aggressive tendencies completely subsided, and he is currently stable. The patients’ timelines and information are shown in Figure 1 and Table 1.

## 3. Discussion

Both cases were characterized by a sudden onset of personality change and cognitive impairment, and colorectal cancer was diagnosed shortly after; the cognitive impairment improved after surgical treatment and chemotherapy. No abnormalities were observed during the brain MRI and CSF cytology; therefore, cognitive impairment due to brain or leptomeningeal metastasis was excluded. Radiation-induced brain injury and steroid dementia syndrome were also excluded, since the patients had never received radiation therapy for the head and neck or immunotherapy with steroids. The possibility of opioid-related adverse effects was unlikely, since they had never been administered narcotic analgesics. The electrolyte levels were also normal; thus, cognitive impairment due to metabolic abnormalities was excluded. In both cases, the clinical symptoms suggested limbic encephalitis, though the patients did not meet the diagnostic criteria for PLE.

PLE is an autoimmune syndrome characterized by memory loss and behavioral and mood changes. In general, PLE can be diagnosed when the four following criteria are met [4]: symptoms of neuropsychiatric dysfunction related to the limbic system; EEG, MRI, or CSF analysis showing characteristic PLE features; cancer diagnosed within four years; and exclusion of other possible diseases. Alternatively, PLE may be diagnosed if autoantibodies specific to cell-surface, synaptic, or onconeural proteins are detected in the serum or CSF [4]. In these cases, the paraneoplastic antibody test results were negative, and neither EEG, MRI, nor CSF analysis met the diagnostic criteria for PLE, as no abnormal findings were observed. Nevertheless, the patients showed characteristic PLE neuropsychiatric symptoms, including rapid cognitive decline, memory loss, and increased violence, and cancers were diagnosed within a year of these symptoms appearing. Both patients met two or more PLE diagnostic criteria; therefore, PLE was probable, and seronegative PLE, which accounts for 10–20% of PLE cases, was suspected. However, the absence of MRI and EEG diagnostic features and CSF lymphocytic pleocytosis and oligoclonal bands contradicted this diagnosis. Therefore, we considered other possible causes.

Three hypotheses may be considered for the cause of cognitive impairment in these cases. The first hypothesis is the gut–brain connection and tumor-induced inflammatory cytokines, since both patients were diagnosed with colorectal cancer. Gut–brain connections can be explained by two concepts: the brain–gut axis, suggesting that changes in brain function such as anxiety, tension, sadness, and anger affect the digestive functions; and conversely, the gut–brain axis, assuming that changes in the intestinal flora can affect brain functions [5]. Both patients were over 60 years of age, and they possibly had microbiota dysfunction due to physiological and age-related intestinal changes. These microbiota changes may have caused sudden personality and emotional alterations in these patients. This is because short-chain fatty acids (SCFAs), gut-derived microbial metabolites, are associated with nuclear factor-κB (NF-κB) activity and regulate proinflammatory immune responses. Studies have shown that microglial cells connect the gut to the brain with G-protein-coupled receptors, which recognize SCFAs, and the gut microbiota can communicate directly with microglia resident in the brain via the vagus nerve [6]. In addition, inflammatory cytokines released from colorectal cancers may influence the central nervous system. Proinflammatory cytokines produced by the tumor, such as IL-1a, IL-1b, IL-6, and TNF-α, can cross the blood–brain barrier (BBB) and migrate to the brain [7]. IL-6 upregulates NMDA receptors, inducing neurotoxicity; this is one of the main mechanisms causing neurodegenerative disorders, including Alzheimer’s disease. In addition, TNF-α crosses the BBB and activates TNF-α receptor 1 in astrocytes, causing hippocampal synaptic alterations and memory impairment.

The second hypothesis is that limbic encephalitis was caused by CD8^+^ T cells. According to a recent report, cell-mediated immunity may induce autoimmune inflammation in the limbic gray matter [8]. This activation is distinct from the CD4^+^ T-cell-mediated humoral immunity to cancer-derived antigens, a typical mechanism of PLE; however, it may cause clinical symptoms similar to those of PLE, such as memory loss, and emotional and behavioral changes. These patients presented clinical symptoms similar to those described in previously reported cases of CD8^+^ T-cell-mediated limbic encephalitis; therefore, this hypothesis can be considered the cause of PLE-like clinical symptoms. Unlike PLE limbic dysfunction mediated by the major histocompatibility complex class II (MHC II) and CD4^+^ T cells, the neurological dysfunction in these patients was possibly induced by CD8^+^ cytotoxic T cells activated via MHC I. The direct interaction of CD8^+^ cells and neurons results in the immediate regulation of neuronal excitability and network activity, possibly causing neuropsychiatric symptoms, including acute symptomatic seizures [8]. This is because CD8^+^ T-cell-derived perforin monomers aggregate to form large voltage-independent poly-perforin channels in the cell membrane, and subsequent ion flux abrogates the transmembrane electrochemical ion gradient and, finally, results in complete electrical silencing and disruption of the cell [8]. Additionally, CD8^+^ T-cell-derived interferon-γ (IFN-γ) has been shown to elevate glutamate excitotoxicity through direct intracellular trans-signaling between IFN-γ and AMPA/kainate receptors [8]. Recent reports suggest that CD8^+^ T-cell activity may be increased or decreased through epigenetic regulation of this immune checkpoint and exhaustion markers in colorectal cancer patients [9,10]. Further studies related to CD8^+^ T-cell activity in the treatment of limbic encephalitis are needed. 

The third hypothesis suggests that the patients’ symptoms could be attributed to somatic mutations in dementia-related genes in cancer patients; however, thorough research on this issue has not been conducted. According to a recent report, the genomic instability of cancer tissues may cause somatic mutations in dementia-related genes, potentially causing the accumulation of neurodegenerating substances such as amyloid beta, hyperphosphorylated tau, and alpha-synuclein [11]. This finding suggests that somatic mutations in cancer patients may be a causative mechanism for dementia, and we think this possibility needs to be considered. As decreased tumor burden can lead to decreased transcription of the dementia-related gene, the reason for the improvement in the cognitive symptoms of the patients after cancer treatment is not that the somatic mutation was reversed; rather, the somatic mutation caused by the tumor tissue did not occur anymore.

After treatment for the underlying tumors, the patients’ progress was positive, and their cognitive function was restored to previous levels. This improvement is in contrast to the typical course of dementia, which is chronic and irreversible, and suggests that a different disease should be suspected as the cause of cognitive decline in these patients. Therefore, in cases of sudden unexplainable decline in cognitive function and personality changes, it is necessary to consider cancer as the cause, as one of the first hypotheses. In addition, we believe that appropriate treatment can help improve the prognosis.

## Figures and Tables

**Table 1:** Patients’ information. CSF—cerebrospinal fluid; DM—diabetes mellitus; HTN—hypertension; K—potassium; Na—sodium; TSH—thyroid stimulating hormone; WBC—white blood cell.

		Case 1 (68/Male)	Case 2 (82/Male)
Past history		HTN (−) DM (−)	HTN (+) DM (−)
Blood chemistry	WBC (/mm^3^) [4800–10,800]	7610	5420
	Na^+^ (mEq/L) [136–145]	133	138
	K^+^ (mEq/L) [3.5–5.1]	4.4	4.27
Thyroid function test	TSH (uIU/mL) [0.55–4.78]	1.564	1.142
	Free T4 (pmol/L) [11.5–22.7]	14.86	13.24
CSF study	Pressure (cmH_2_O) [5–20]	14	12
	WBC (/mm^3^) [0–5]	3	0
	Glucose (mg/dL) [50–80]	68.0	61
	Protein (mg/dL) [15–45]	79.1	74
Diagnosed cancer		Rectal cancer (T3N0, stage IIA)	Ascending colon cancer (T3N1b, stage IIIB)

## Data Availability

Clinical data may be provided to the editors upon appropriate request.

## References

[1] Wefel J.S., Kesler S.R., Noll K.R., Schagen S.B. (2015). Clinical characteristics, pathophysiology, and management of noncentral nervous system cancer-related cognitive impairment in adults. CA Cancer J. Clin..

[2] Gultekin S.H., Rosenfeld M.R., Voltz R., Eichen J., Posner J.B., Dalmau J. (2000). Paraneoplastic limbic encephalitis: Neurological symptoms, immunological findings and tumour association in 50 patients. Brain.

[3] Pelosof L.C., Gerber D.E. (2010). Paraneoplastic syndromes: An approach to diagnosis and treatment. Mayo Clinic Proceedings.

[4] Graus F., Titulaer M.J., Balu R., Benseler S., Bien C.G., Cellucci T., Cortese I., Dale R.C., Gelfand J.M., Geschwind M. (2016). A clinical approach to diagnosis of autoimmune encephalitis. Lancet Neurol..

[5] Emeran A.M., Kirsten T., Arpana G. (2015). Gut/brain axis and the microbiota. J. Clin. Investig..

[6] Wang Y. (2018). The Gut-microglia connection: Implications for central nervous system diseases. Front. Immunol..

[7] Timothy R.S., Sarkis K.M. (2015). Control of brain development, function, and behavior by the microbiome. Cell Host Microbe.

[8] Ehling P., Melzer N., Budde T., Meuth S.G. (2015). CD8^+^ T cell-mediated neuronal dysfunction and degeneration in limbic encephalitis. Front. Neurol..

[9] Yang R., Cheng S., Luo N., Gao R., Yu K., Kang B., Wang L., Zhang Q., Fang Q., Zhang L. (2019). Distinct epigenetic features of tumor-reactive CD8^+^ T cells in colorectal cancer patients revealed by genome-wide DNA methylation analysis. Genome Biol..

[10] Nair S.V., Saleh R., Toor S.M., Taha R.Z., Ahmed A.A., Kurer M.A., Murshed K., Nada M.A., Elkord E. (2020). Epigenetic Regulation of Immune Checkpoints and T Cell Exhaustion Markers in Tumor-Infiltrating T Cells of Colorectal Cancer Patients. Epigenomics.

[11] Kim Y.C., Jeong B.H. (2020). Identification of somatic mutations in dementia-related genes in cancer patients. Curr. Alzheimer Res..

